# Perceptions of Child Abuse as Manifested in Drawings and Narratives by Children and Adolescents

**DOI:** 10.3389/fpsyg.2020.562972

**Published:** 2021-01-14

**Authors:** Limor Goldner, Rachel Lev-Wiesel, Bussakorn Binson

**Affiliations:** ^1^Sagol Lab for Children at Risk, The Emili Sagol Creative Arts Therapies Research Center, Faculty of Social Welfare and Health Sciences, School of Creative Arts Therapies, University of Haifa, Haifa, Israel; ^2^FAA-Emili Sagol Creative Arts Research and Innovation for Well-Being Center, Faculty of Fine and Applied Arts, Chulalongkorn University, Bangkok, Thailand

**Keywords:** child abuse, drawings, narratives, disclosure, art therapy

## Abstract

Child abuse is an underreported phenomenon despite its high global prevalence. This study investigated how child abuse is perceived by children and adolescents as manifested in their drawings and narratives, based on the well-established notion that drawings serve as a window into children’s mental states. A sample of 97 Israeli children and adolescents aged 6–17 were asked to draw and narrate what child abuse meant to them. The drawings and narratives were coded quantitatively. The results indicated that participants did not perceive a distinction between abuse and violence and referred to them interchangeably. Almost half of the participants focused on emotional abuse. The most frequent type of abuse within the family was between parents and children, and the most frequent abuse outside the family was peer victimization. Most of the drawings were figurative and realistic and half of the drawings included words suggestive of the participants’ attempts to be heard and fully understood. The vast majority of drawings did not include the figure of the artist, about a third of the drawings employed dissociative techniques (i.e., included positive objects, were unrelated to abuse, used words alone, or did not follow the instructions), and almost half of the narratives were dissociative or characterized by negative resolution, describing feelings such as sadness, humiliation, and loneliness. These findings suggest the emotional pain associated with the abuse or violence and the use of dissociative mechanisms to bypass the pain. The findings are discussed in light of the literature on children’s disclosure.

## Introduction

Child abuse, which is defined as any act or series of acts of commission or omission by a parent, other caregivers, or a stranger that results in harm, the potential for harm, or the threat of harm to a child (Daphna-Tekoah et al., 2019), is a worldwide phenomenon (Wildeman et al., 2014; Stoltenborgh et al., 2015). In addition, violence against children is hugely prevalent, with more than half of all children worldwide annually exposed to violence in all its forms (Lev-Wiesel et al, 2018). A nationwide study on American youth’s exposure to violence covering conventional crime, child maltreatment, peer and sibling offenses, sexual assault, witnessing, and indirect exposure to violence, and internet offenses revealed that 60% reported at least one form of direct exposure to violence in the past year (Finkelhor et al., 2005). A recent epidemiological survey in Israel that focused on the level of youth’s exposure to the different forms of abuse revealed that 52% reported experiences of abuse at least once during their lifetime (Lev-Wiesel et al., 2019). These victims often suffer multiple and varied forms of abuse (Clemmons et al., 2003; Arata et al., 2005; Finkelhor et al., 2007), within and outside the family, by adults or peers (Oriol et al., 2017). Studies report a higher prevalence of bullying in boys than in girls (Hong and Espelage, 2012). Girls were found to be exposed to a higher incidence of emotional abuse by peers (Seals and Young, 2003). These findings demonstrate that every second child is exposed to any injury.

Yet, despite the high prevalence of child abuse, whether inflicted by a family member, a stranger, or a peer, less than 10% of the children ever disclose the abuse, and about 70% of children are reluctant to disclose it to professionals (Lev-Wiesel and First, 2018). Thus, the prevalence of abuse and its enduring harm and the gap between the prevalence of child maltreatment and disclosure underscores the need to document how children and youth perceive abuse and which abusive behaviors are required to be disclosed to adults according to children and adolescents. Although studies have examined perceptions of child maltreatment among pediatricians (Al-Moosa et al., 2003), social workers (Ashton, 2010), law enforcement officers, educators, and mental health and healthcare professionals (Eisikovits et al., 2015), as well as among teachers (Gamache Martin et al., 2010), much less work has been done on children’s and teens’ perceptions (Lev-Wiesel et al., 2020).

Recently, Lev-Wiesel et al. (2020) compared children’s and parents’ perceptions of child maltreatment and abuse. The findings showed that both parents and children considered sexual, emotional, and physical maltreatment, as well as neglect, as more damaging than child labor. However, children perceived emotional abuse to be significantly more severe than did their parents, and the parents, unlike their children, rated sexual abuse as the most damaging. Built on these preliminary efforts, the current study aimed to shed light on the ways children and adolescents from the general population perceive child abuse, as manifested in their drawings and narratives. Unfolding children’s perception of the abuse may assist therapists, parents, and educators in understanding the ways children experience this disturbing reality. Moreover, if clinicians, parents, and educators want to encourage children to disclose the abuse, they need to comprehend when children perceive violent situations as either abusive or non-abusive.

### Drawings and Narratives as a Window on Abuse

Empirical and clinical work in art therapy and allied fields has shown that drawings enable the expression of hidden or repressed thoughts and feelings in a relatively fast and straight forward way (Lev-Wiesel and Shvero, 2003; Jacobs-Kayam et al., 2013; Woolford et al., 2013; Snir et al., 2020). Regarding children, researchers and therapists posited that drawings could shed light on children’s internal and outer worlds and their perception of their families and parents in a way that communicates feelings and ideas of their environment (Goldner and Scharf, 2012). According to the authors, colors, shapes, and motifs can all represent the unconscious, ideas, distressful feelings and thoughts, concerns, and worries, adding layers of meaning to verbal content (Lev-Wiesel and Liraz, 2007; Goldner and Scharf, 2012; McInnes, 2019). Recently, researchers in social sciences have suggested that drawings serve as a powerful adjunct to traditional data collection approaches as they advance researchers’ understanding regarding individuals’ perceptions of pathology and well-being (Huss et al., 2012; Boden et al., 2019; Broadbent et al., 2019).

Furthermore, prior studies indicated that drawings could encourage the verbalization of traumatic experiences. Enabling the drawer to become a spectator to his or her negative experience, drawings facilitate the production of a rich, detailed description composed of emotions and facts from a somewhat detached and protected perspective (e.g., Dayton, 2000; Lev-Wiesel and Liraz, 2007). In this respect, clinical and empirical evidence indicates that children’s drawings, particularly those done during a time of crisis, can overcome children’s language limitations (Lev-Wiesel, 1999; Dayton, 2000; McInnes, 2019). For instance, Cohen and Ronen (1999) found that children who had experienced parental divorce reflected their negative experiences in their family drawings. Drawings were shown to enable sexually abused children to provide more details and speak more coherently about what had happened to them, thus enhancing these children’s testimonies (Macleod et al., 2013; Katz et al., 2014, 2018).

Similarly, narratives and the process of generating information through storytelling can shed light on the way meaning is constructed and the ways humans experience the world (Lucius-Hoene and Deppermann, 2000). With traumatic memories, therapists’ consensus is that autobiographical memory of the traumatic events leads to a fragmented narrative (Midgley, 2002; Lev-Wiesel, 2008). However, the reconstruction of a rich, coherent autobiographical memory is essential for healing (Van Minnen et al., 2002). Thus, for example, in narrative exposure therapy, children are requested to describe in detail what happened to them, attentively focused on sounds, smells, feelings, thoughts, and recall. According to researchers, children who have experienced multiple and very severe traumatic events should be treated with narrative approaches. It has been suggested that by habituation to the negative emotions relating to the painful memories, the symptoms of distress might decrease (Neuner et al., 2008; Ruf et al, 2010).

In the current study, we focused on the ways children and youth perceive child abuse through both drawings and narratives. The use of an integrated approach that involves visual, non-verbal, and verbal methods may serve as a venue exploring the multiplicity and complexity of children’s experience (Lev-Wiesel and Liraz, 2007). Thus, each modality substantiated and enriched the other (Boden et al., 2019).

## Materials and Methods

### Participants and Procedure

Ninety-seven children and adolescents from the general population (41.2% girls, *n* = 57, mean age = 9.38, range 6.00–17.00; *SD* = 2.75) were recruited to this study. The study was a part of a large project conducted in a school setting, which aimed to learn about the perception of child abuse among children and youth (for the demographic variables, see Table 1). After receiving ethical approval from the Committee to Evaluate Human Subject Research of the Faculty of Health Sciences and Social Welfare of the University of Haifa (Number: 122/15), parents received letters explaining the study aims and risks and were asked to sign informed consent forms. Upon parents’ approval, research assistants introduced the project to children and adolescents and asked them to draw “what is child abuse for you” on an A4 (21 × 29.7 cm) sheet of white drawing paper using a set of 12 crayons and a pencil. There was no time limit. They were then asked to provide a written narrative in Hebrew recounting the drawings. Some of the narratives have been translated into English for the purpose of writing this article. Children were not asked to paint or describe a specific experience that had happened to them. However, in case of discomfort, children were assured of getting preliminary assistance from the research team by debriefing their emotions and referring them to school counselors. Participants were assured of the confidentiality of their drawings and narratives. Parents were assured that the analysis of the drawings and the narratives would be conducted as a group and that the drawings will be used for research purposes.

**TABLE 1:** Participants’ demographics.

Variable		Percentage (frequency)
**Gender**		
	Boys	58.8% (57)
	Girls	41.2% (40)
**Ethnicity**		
	Jewish	83.5% (81)
	Arabs	16.5% (16)
**Family status**		
	Married	85.6% (83)
	Single	14.4% (14)
**SES**		
	High	18.6% (89)
	Medium	51.5% (50)
	Low	29.9% (29)

### Measures

#### Drawing and Narrative Coding

The current study applied principles of a multimodal method (for drawings and narratives), the relational mapping interview, which was developed by Boden et al. (2019) to understand the relational context of distress, in this specific case abuse, as manifested in drawings and narratives by children. Incorporating drawing into interpretative phenomenological analysis (IPA) design provides a vehicle through which the children can communicate their experiences and perceptions. Drawing activates several senses simultaneously, providing data, which in turn sheds light on and reveals the phenomenon. Combining drawings with narratives about what is drawn results in richly nuanced visual and verbal accounts of relational experience. The analysis, therefore, incorporated the visual reflective method of drawing and the narrative into the IPA.

Furthermore, there is extensive mental health, self-defining memory, and art therapy literature on art-based assessments that uses an objective, quantitative approach to measure the frequency of items depicted in various types of drawings and narratives that have been considered indicators of possible emotional problems in non-clinical and clinical populations. These tests incorporate a multiple-sign approach to assist researchers and art therapists in understanding individuals’ mental world (Gantt, 2004; Goldner et al., 2018).

Following these methodological approaches, and based on the literature on child abuse and dissociation (Hetzel and McCanne, 2005; Lev-Wiesel, 2005; Nijenhuis et al., 2010), trauma resolution (Main and Hesse, 1990; Scharf and Shulman, 2006), and self-defining memories (Thorne and McLean, 2002), a quantitative protocol that combined the pictorial features of the drawings with the themes of the narrative was developed. The first two authors carefully examined the drawings and the narratives, identifying common features align with the literature (for the protocol categories and indicators, see Table 2).

**TABLE 2:** Characteristics of the drawings and narratives.

Abuse/violent scene included	1. Yes	86.6 (84)
	2. No	13.4 (13)
Abuse within the family	1. Yes	51.5 (50)
	2. No	48.5 (47)
The relationship between the aggressor and the victim	1. Siblings	18.6 (18)
	2. Parent-child	16.5 (16)
	3. Spouses	5.1 (5)
	4. Other: natural disaster, terror, car accident, crime	12.4 (12)
	5. friends	18.6 (18)
	6. The entire family (including the child)	10.3 (10)
	7. Adult (not parent)-child	5.1 (5)
	8. No abuse	13.4 (13)
Type of the abuse	1. Physical	32.0 (31)
	2. Sexual	4.1 (4)
	3. Emotional	40.2 (39)
	4. Mixed	8.2 (8)
	5. Not specified	15.5 (15)
Does the drawing include the artist?	1. Yes	18.6 (18)
	2. No	81.4 (79)
Type of drawing	1. Figurative/realistic	85.5 (83)
	2. Expressive/metaphoric	12.4 (12)
	3. No drawing	2.1 (2)
Words included	1. Yes	47.4 (46)
	2. No	52.6 (51)
Does the drawing match the child’s chronological age?	1. Yes	70.1 (68)
	2. No	29.9 (29)
Is there any physical contact in the painting between the victim and the aggressor (between the depicted objects)?	1. Yes	30.9 (30)
	2. No	69.1 (67)
Victim size	1. Tiny	18.6 (18)
	2. Normal	50.5 (49)
	3. Exaggerated	3.1 (3)
	4. Not depicted	27.8 (27)
Aggressor size	1. Tiny	11.3 (11)
	2. Normal	48.5 (47)
	3. Exaggerated	12.4 (12)
	4. Not depicted	27.8 (27)
Does the drawing include signs of aggression, beatings, punching, or threats?	1. Yes	61.9 (60)
	2. No	38.1 (37)
Does the drawing include any signs of injury, blood, pain, or tears?	1. Yes–physical injury	15.5 (15)
	2. Yes–emotional injury	29.9 (29)
	3. Yes–both	1.0 (1)
	4. No	53.6 (52)
Is the painting pre-schematic, limited, blocked human figures, primitive figures, correspond to ages 4–5?	1. Yes	40.2 (39)
	2. No	59.8 (58)
Level of vitality referring to emotional investment in drawings reflected in embellishment, colorfulness, and details	1. Low level	36.1 (35)
	2. Medium level	33.0 (32)
	3. High level	30.9 (30)
Dissociation within the drawing, detached, does not address the pain.	1. Yes	61.9 (60)
	2. No	38.1 (37)
What role does the artist play in the narrative?	1. Victim	19.8 (19)
	2. Aggressor	3.1 (3)
	3. Bystander	6.3 (6)
	4. Mixed	5.2 (5)
	5. No specific role	65.6 (63)
Dissociation within the narrative, the narrative does not concentrate on the violence, detached, does not address the pain?	1. Yes	53.1 (51)
	2. No	46.9 (45)
Narrative organization	1. Coherent, organized	43.7 (42)
	2. Restricted	37.5 (36)
	3. Chaotic, Flooded	18.8 (18)
Central theme	1. Child’s anxiety, dread, helplessness, and powerlessness	13.5 (13)
	2. Child’s humiliation, sadness, loneliness, guilt and shame	45.8 (44)
	3. Mother’s helplessness, loneliness, and sadness	4.2 (4)
	4. The child gets stronger as a result of the incident	3.1 (3)
	5. Narrow, avoidant description without emotions, no reference to the abuse	33.4 (32)
Narrative resolution	1. Positive	17.7 (17)
	2. Negative	51.0 (49)
	3. No solution, neutral	31.3 (30)
Does the drawing coincide with the narrative?	1. Yes	85.4 (82)
	2. No	14.6 (14)

#### Drawings

The drawings coding system included indicators regarding the content and the style of the drawing. These two dimensions are considered central in investigating art products (Gantt, 2004; Matto and Naglieri, 2005) or art perception (Augustin et al., 2011). Content indicators related to the abuse/violence in the drawings were coded as follows: (1) depiction of a violent/abusive scene in the drawing (yes/no), (2) abuse within the family (yes/no), (3) the relationship between the aggressor and the victim [siblings, parent–child, spouses, other, terror, crime, accident, friends, entire family (including the child), adult (not a parent)–child outside of the family, no abuse], (4) the type of abuse/violence (physical, sexual, emotional, mixed, not specified), and (5) whether the artist is depicted in the drawing (yes/no).

The drawing style was coded using the following indicators: (1) drawing type (figurative/realistic, expressive/metaphoric, no drawing), (2) whether the drawing included words (yes/no), (3) whether the drawing matched the child’s chronological age (yes/no), (4) physical contact between the victim and the aggressor (between the depicted objects) (yes/no), (5) the size of the victim (tiny—the figure is small, about 2 cm, and occupies a small part of the page space; normal—the figure is proportional to the space of the page and the other figures; exaggerated—the figure occupies most of the page space and leaves no room for other objects or its size is not proportional to the other figures; not depicted), (6) the size of the aggressor (tiny, normal, exaggerated, not depicted), (7) overall impression of helplessness in the drawing (yes/no), (8) presence of aggressive symbols manifested in beatings, punches, or threats (yes/no), (9) depictions of injury manifested in blood, pain, tears to represent physical, emotional injury; mixed injuries; none, (11) whether the drawing was pre-schematic, limited, blocked human figures, primitive figures corresponding to ages 4–5 (yes/no), (12) level of vitality, referring to emotional investment in the drawing reflected in embellishment, colorfulness, and details (low, medium, high), (13) whether the drawing included dissociation, was detached, did not address the pain (i.e., included positive objects such as flowers, hearts, or non-related objects; contained only words; or did not follow the instructions, thus bypassing the painful meaning of the abusive scene) (yes/no).

#### Narratives

The coding of the narratives’ search for coherence was based on methods described in the literature on trauma resolution (i.e., the extent to which individuals regain emotional and behavioral control and understand how trauma has affected both their inner world and their behavior) (Spooner and Lyddon, 2007; Saltzman et al., 2013). While resolved traumatic memories narratives are rich, specific, and integrative, reflecting individuals’ ability to merge affect and cognition in an optimal manner, chaotic unresolved traumatic memories are confused, excessively detailed, and contradictory and contain themes of failure and subsequent humiliation by caregivers, and restricted dismissing narratives are emotionless, over general, and convey a vague and diffuse sense of self and others (Goldner and Scharf, 2017). The narratives were coded using the following indicators: (1) the role of the artist in the drawing (victim, aggressor, bystander, mixed, no specific role), (2) presence of dissociation (i.e., the narrative does not concentrate on the violence or abuse, is detached, does not address the pain), (3) narrative organization coherent/organized, restricted—the inability to describe the event, chaotic/flooded—incoherent, flooded with detail description (3) central theme (child’s anxiety, dread, helplessness, and powerlessness/child’s humiliation, sadness, loneliness, guilt, and shame/mother’s helplessness, loneliness, and sadness/the child getting stronger as a result of the incident/narrow avoidant description without emotion, no reference to the abuse), (4) resolution of the narrative (positive, negative, no solution, neutral), and (5) whether the content of the drawing coincided with the content of the narrative (yes/no). The drawings and narratives were coded together by the first two authors, who are experts in art-based assessments. Disagreements were resolved through consensus.

## Results

### Preliminary Analysis

A series of chi-square analyses was performed to identify differences in drawings and narratives across boys and girls and age groups. Data indicated that boys depicted more tiny victims (41.9%) compared to girls (12.8%), [χ^2^(2) = 8.99, *p* = 0.01] and used more preschematic drawings (53.8%) compared to girls (31.6%), [χ^2^(1) = 4.76, *p* = 0.03]. Girls depicted more vital drawings (42.1%) compared to boys (15.1%), [χ^2^(2) = 3.10, *p* = 0.02]. However, they produced more negative narratives (66.7%) compared to boys (30%), [χ^2^(2) = 12.44, *p* = 0.00]. Elementary-school-aged children depicted more helplessness drawings (69.6%) compared to adolescents (45.9%), [χ^2^(1) = 3.92, *p* = 0.04] and produced more neutral resolution (37.1%), [χ^2^(2) = 6.90, *p* = 0.03] compared to adolescents, who generated more positive narratives (34.8%).

Children from families with two parents who were not divorced used less dissociative mechanisms in their drawings (31.4%) compared to children from divorced or single-parent families (78.6%), [χ^2^(1) = 11.2, *p* = 0.00] and their drawings coincided more with the narrative (92.7%) compared to children from divorced or single-parent families (42.9%), [χ^2^(1) = 23.88, *p* = 0.00]. Children from families with high socioeconomic status tended to depict more abuse/violence scenes within the family (88%) compared to children from families with low (24.2%) or median (57.9%) socioeconomic status [χ^2^(2) = 14.50, *p* = 0.00]. The most frequent type of abuse/violence depicted by children from families with high socioeconomic status was between siblings (50%), while the most frequent type of abuse/violence depicted by children from families with low and medium socioeconomic status was between friends (31, 14%, respectively) [χ^2^(14) = 33.69, *p* = 0.00]. Adolescents’ drawings incorporated more emotional injury symbols (47.8%) compared to drawings of elementary-school-aged children, which incorporated more symbols of physical injury (58.1%), [χ^2^(3) = 8.53, *p* = 0.04].

### Characteristics of the Drawings and the Narratives

**FIGURE 1:**
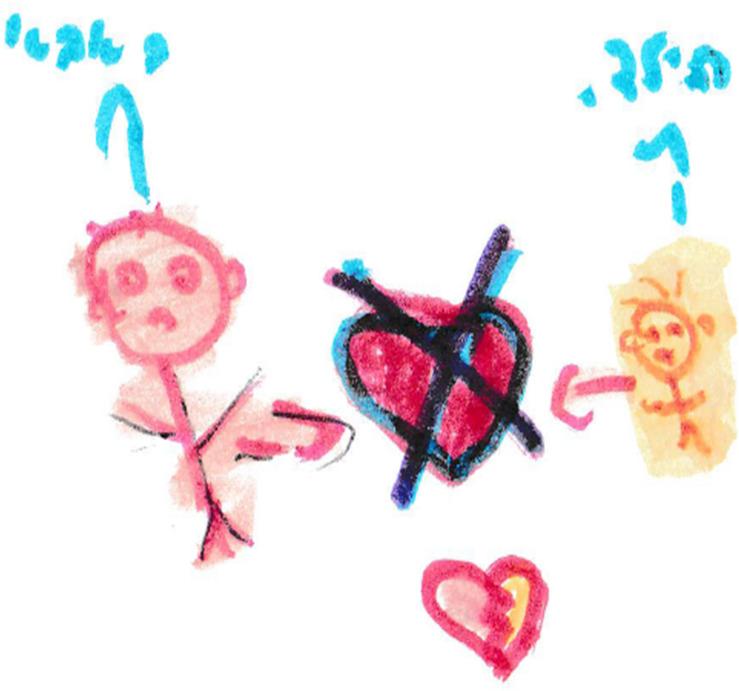
An example of drawing and narrative of parent-child abuse drawn by a child. “A father and son. The father hit the son and then the son feels that the father doesn’t love him. The child has a broken heart and feels a lack of love”.

**FIGURE 2:**
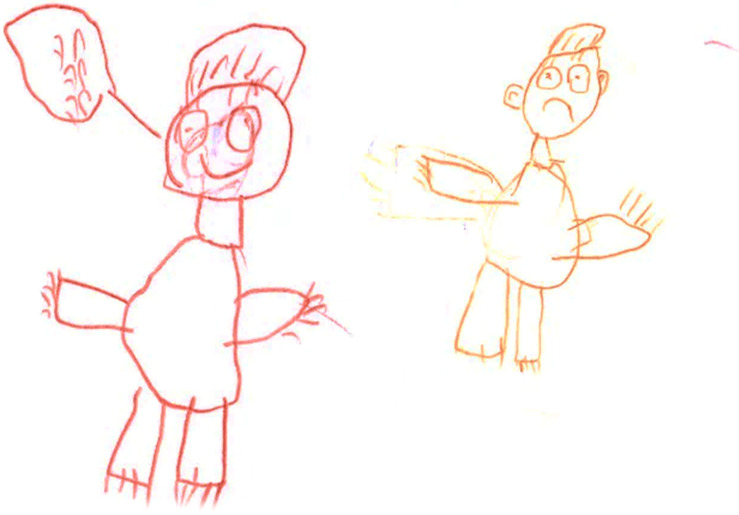
An example of a drawing of peer abuse drawn by a child. “One child made fun of me because I am fat and then all the children in the class made fun of me, and every day I got sick with a different disease. All the children in the class realized it was wrong, and said they were sorry. They gave me candies to apologize”.

**FIGURE 3:**
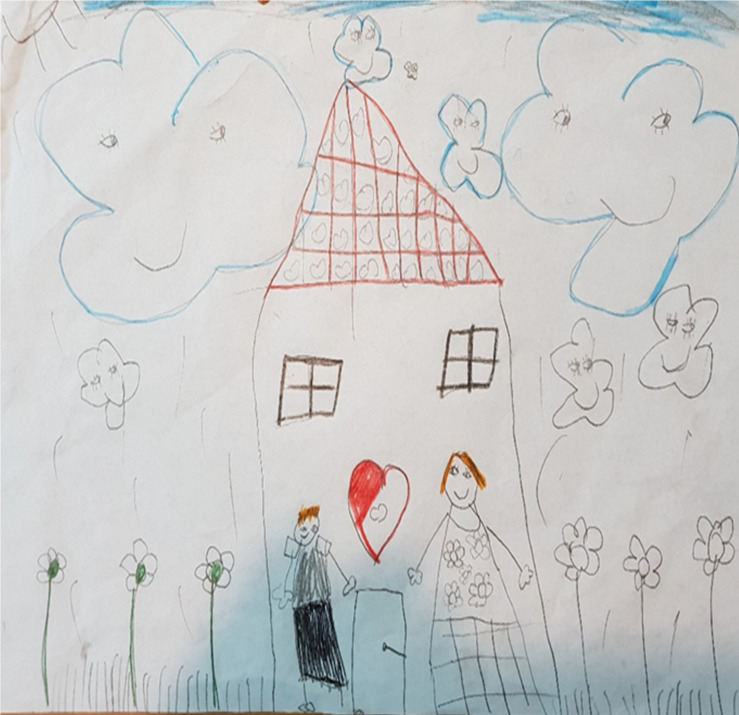
An example of dissociation within the drawing, drawn by a child. “A family of clouds. This is mom and this is dad and here are flowers and more flowers, and they go outside and pick flowers”.

**FIGURE 4:**
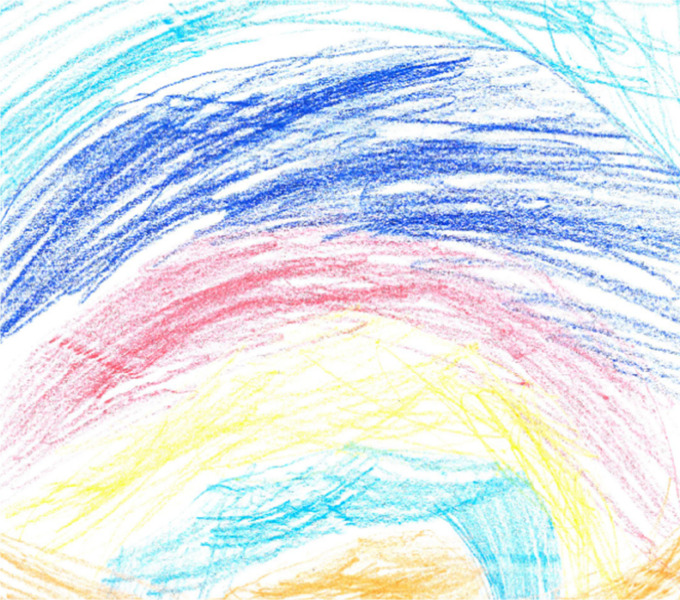
An example of dissociation within the drawing and narrative created by a child. The participant refused to follow instructions to draw what he thought was child abuse. He/she asked if he/she could draw the opposite of violence and something that appealed to him/her. He/she chose to draw a rainbow and said that his/her painting was called a “Rainbow of Peace”.

As shown in Table 2, most of the drawings were either figurative or realistic (85.6%) and included an abusive/violent scene (86.6%). Almost half of the drawings focused on emotional abuse (40.2%). About half of the participants depicted abuse within the family (51.5%), and the most frequent type of violence depicted was between parents and children (16.5%) or between siblings (18.6%). The most frequent category of violence outside the family was between friends (18.6%) (see Figures 1, 2). The vast majority of participants did not include themselves in the drawings (81.4%). More than half of the drawings did incorporate symbols of aggression (61.9%) and almost half of the drawings included injury symbols (46.4%). About a third of the drawings involved dissociative techniques (61.9%). About half of the drawings were preschematic (40.2%) and included words (47.4%) (for examples, see drawings and narratives in Figures 3, 4). Most of the narratives were consistent with the drawings (85.4%). Approximately a quarter (28.1%) of the narratives described the child as exposed to direct violence as either victim or aggressor, while in the rest of the narratives the child did not have a specific role or was exposed to indirect violence as a bystander. About half of the narratives were coherently organized (43.7%), whereas the rest were either restricted (37.5%) or chaotic (18.8%), revealing dissociation, distress, sadness, and loneliness.

### Associations Between Type of Abuse and Drawings and Narrative Characteristics

**FIGURE 5:**
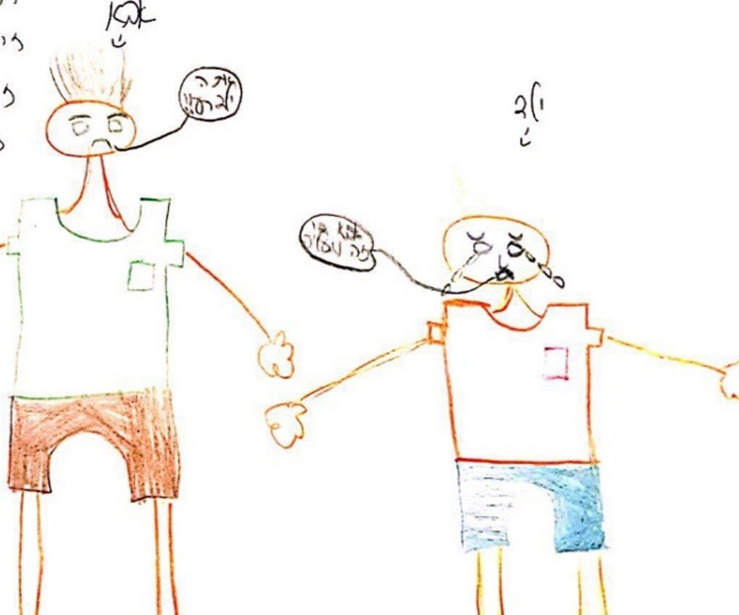
An example of a drawing with symbols of hurt drawn by a child. “Dad says ‘You’re a bad boy’ and the boy cries and says ‘Dad, enough.’ This is emotional abuse, not physical, I’ve drawn emotional abuse.” The child in the drawing is not the artist; it is meant to represent any child.

**FIGURE 6:**
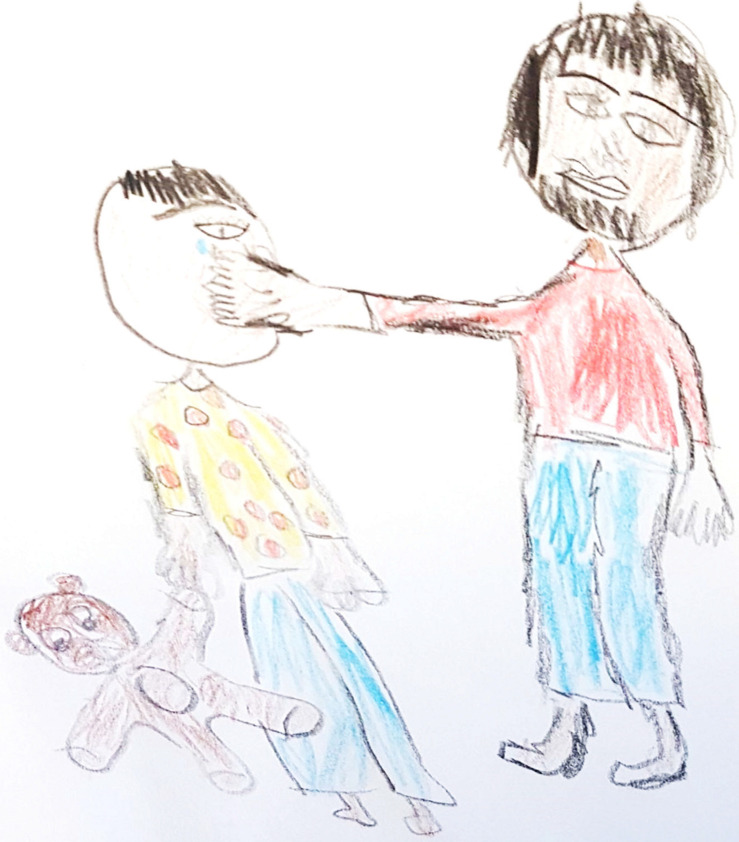
An example of a drawing with aggression symbols and a helplessness narrative created by an adolescent. “I painted a dad who beats his kid, I don’t know why. I painted a bear, I don’t know, it doesn’t matter to the teddy bear. You see that the father is sad and the boy is crying, that hurts him. Usually, domestic violence doesn’t really happen, it’s more school violence. In this house, for example, one of the parents got upset or something, or was angry and said something insulting: I don’t want to bring you up, why were you born? After this quarrel they don’t really mean it, it’s just, you don’t really have to do it because after that you can see the child lying in bed and doing nothing. This once happened to me, I’m sorry to say”.

Chi-square analyses were conducted to identify differences in the participants’ drawings and narratives according to the type of abuse/violence. As can be seen in Table 3, the analyses revealed differences in relation to the presence of a violent scene, physical contact between the victim and the aggressor, the inclusion of aggressive symbols, and dissociation within the drawings and narratives. In particular, violent scenes, physical contact between the victim and the aggressor, and aggressive symbols were present in drawings containing multiple forms of abuse or physical abuse as compared to only emotional or only sexual abuse (for examples, see drawings in Figures 5, 6). More dissociations within the drawings and narratives were depicted when the type of abuse was not specified, as compared to sexual, physical, or emotional abuse (see Table 3).

**TABLE 3:** Associations between type of abuse and drawings and narrative characteristics.

		Abuse type						
Indicator	Category	Physical (*n* = 31)	Sexual (*n* = 4)	Emotional (*n* = 39)	Mixed (*n* = 8)	Not specified (*n* = 15)	*df*	χ*^2^*
Abuse included	1. Yes (*n* = 68)	27	2	28	8	3	4	26.47
	2. No (*n* = 29)	4	2	11	0	12		*p* < 0.001
Is there any physical contact in the drawing between the victim and the aggressor (between the objects)?	1. Yes (*n* = 30)	16	1	6	5	2	4	16.59
	2. No (*n* = 67)	15	3	33	3	13		*p* < 0.01
Does the drawings include signs of aggression, beatings, punching?	1. Yes (*n* = 60)	26	1	24	6	3	4	20.40
	2. No (*n* = 37)	5	3	15	2	12		*p* < 0.001
Dissociation within	1. Yes (*n* = 37)	12	2	9	2	12	4	15.71
the drawing	2. No (*n* = 60)	19	2	30	6	3		*p* < 0.01
Dissociation within the narrative	1. Yes (*n* = 45)	14	2	12	5	12	4	13.38
	2. No (*n* = 51)	17	2	27	3	2		*p* < 0.01
								

### Associations Between Drawings and Narratives

Chi-square analyses were conducted to identify the associations in the participants’ drawings and narratives. The analyses revealed correlations between the extent to which the drawing was consistent with the narrative and the presence of violent scenes or aggressive symbols. More violent scenes and aggressive symbols were found in the drawings when the narrative was consistent with the drawing compared to narratives that departed from the drawing (see Table 4). Correlations were also found between the presence of dissociative narratives and dissociative drawings that included the self in the drawings and scenes of abuse. In almost all the dissociative narratives, self-figures were not included as compared to the non-dissociative narratives. Almost two-thirds of the dissociative narratives were associated with dissociative drawings that did not include abusive or violent scenes (see Table 5). Finally, the narrative resolution was correlated with the presence of injury or the role of the self-figure in the drawing. Specifically, more symbols of emotional or physical injury were present in both positive and negative narrative resolutions compared to narratives with a neutral resolution. Similarly, more children depicted themselves as victims or aggressors when the resolution was either negative or positive as compared to narratives with neutral resolutions (see Table 6). Finally, children whose narratives discussed direct exposure to violence tended to include the artist in the drawing, to depict a higher rate of violent scenes within the family, and to depict helplessness in the drawings. By contrast, none of the children or adolescents who narrated stories on indirect exposure to violence depicted the artist in the drawings. These children and adolescents also depicted a higher rate of violent scenes out of the family or no violent scenes in their drawings, and a smaller percentage of their drawings depicted helplessness (see Table 7).

**TABLE 4:** Associations between drawing characteristics and match between the drawing and the narrative.

		Drawing coincides with the narrative (*n* = 82)	Drawing does not coincide with the narrative (*n* = 14)	*Df*	χ*^2^*
Abuse included	1. Yes (*n* = 68)	63	5	1	9.79
	2. No (*n* = 29)	19	9		*p* < 0.01
Does the painting include signs of aggression, beatings, punching?	1. Yes (*n* = 60)	56	4	1	8.05
	2. No (*n* = 36)	26	10		*p* < 0.01

**TABLE 5:** Associations between drawing characteristics and dissociation within the narrative.

		Dissociation within the narrative (*n* = 45)	No dissociation within the narrative (*n* = 51)	*df*	χ*^2^*
Abuse included	1. Yes (*n* = 68)	27	41	1	4.81
	2. No (*n* = 28)	18	10		*p* < 0.05
Dissociation within the drawing	1. Yes (*n* = 36)	27	9	1	18.30
	2. No (*n* = 60)	18	42		*p* < 0.001
Does the drawing include the artist?	1. Yes (*n* = 18)	4	14	1	5.41
	2. No (*n* = 78)	41	37		*P* < 0.05

**TABLE 6:** Associations between drawing characteristics and narrative resolution.

		Narrative resolution				
Indicator	Category	Positive resolution (*n* = 17)	Neutral resolution (*n* = 30)	Negative resolution (*n* = 49)	*df*	χ*^2^*
Does the drawing include any signs of injury, blood, pain, or tears?	1. Yes–physical injury (*n* = 15)	5	2	8	6	15.42
	2. Yes–emotional injury (*n* = 29)	3	6	20		*p* < *0.05*
	3. Yes—both (*n* = 1)	1	0	0		
	4. No (*n* = 51)	8	22	21		
What role does the artist play in the narrative?	1. Victim (*n* = 19)	8	3	8	8	30.71
	2. Aggressor (*n* = 3)	0	2	1		*p* < 0.001
	3. Observer (*n* = 6)	4	1	1		
	4. Mixed (*n* = 5)	0	4	1		
	5. No role (*n* = 63)	5	20	38		

**TABLE 7:** Associations between direct and indirect violence in narratives and drawings characteristics.

		Direct violence (*n* = 27)	Indirect violence (*n* = 69)	*df*	χ*^2^*
Abuse within the family	1. Yes (*n* = 50)	16	33	1	5.03
	2. No (*n* = 46)	11	36		*p* < 0.05
Does the drawing include the artist?	1. Yes (*n* = 18)	18	0	1	56.68
	2. No (*n* = 78)	9	69		*p* < 0.001
Helplessness drawing	1. Yes (*n* = 50)	19	31	1	5.03
	2. No (*n* = 46)	8	38		*P* < 0.05

## Discussion

The current study examined how children and adolescents from the general population perceive child abuse as reflected in drawings and narratives. In general, the findings indicated that the children did not perceive a distinction between abuse and violence and referred to them interchangeably, referring equally to intrafamilial and extrafamilial scenes of child abuse and violence. The most frequent form of abuse depicted by the participants was emotional abuse. This is consistent with previous findings indicating a higher prevalence of emotional abuse reported by children and adolescents than other forms of abuse (Lev-Wiesel et al., 2019). Because emotional abuse often accompanies other forms of abuse, whether intrafamilial or extrafamilial, children and adolescents may be more familiar with it. It is consistent with previous findings showing that many children experience and engage in hostility, teasing, bullying, yelling, criticism, rejection, and ostracism, whether as indirect victims or observers, as victims, or as perpetrators (Nansel et al., 2001; Modecki et al., 2014; Stark, 2015).

Although violent engagement between peers or siblings is common (Nansel et al., 2001; Tucker et al., 2013; Modecki et al., 2014; Wolke et al., 2015), the participants here did not appear to consider these interactions as acceptable but rather as abusive/violent acts, in which there were clear roles of victim and perpetrator. These imbalanced situations can exacerbate feelings of embarrassment, humiliation, sadness, loneliness, and helplessness, which were reflected here in the children’s powerlessness drawings and narratives, as well as in their restricted and chaotic narratives, which may imply children’s difficulties in reflecting and resolving the abuse (Main and Hesse, 1990; Scharf and Shulman, 2006). This raises the more general question of the impact of exposure to violence—whether domestic, in the community, or in the media—on children and how it shapes their views of the world and themselves, their ideas about the meaning and purpose of life, their expectations for future happiness, and their moral development (Cummings et al., 2009).

Most of the drawings communicated the abusive/violent experience realistically through the use of symbols of aggression, injuries, and words, particularly the drawings that depicted physical abuse or multiple forms of abuse. This finding lends weight to previous studies indicating that drawings encourage disclosure of disturbing content that can elevate distress by bypassing dissociative mechanisms (Amir and Lev-Wiesel, 2007). The fact that none of the participants avoided or refrained from completing the assignment, but some employed various graphical techniques such as receding themselves from the situation by seldom drawing themselves within the abusive scene, avoiding taking a specific role in the event, or distancing the aggressor from the victim, may indicate an attempt to protect themselves from the troubling subject and its associated negative feelings by dissociative mechanisms. Furthermore, despite the high prevalence of sexual assault among children (Oriol et al., 2017; Lev-Wiesel et al, 2018), children and adolescents rarely drew this type of assault.

Obviously, some of the children and adolescents could have served as the aggressors, perpetrators of bullying, or bystanders (Nansel et al., 2001); however, using these techniques, as well as adding positive objects such as flowers or hearts, non-related objects, or words, may also reflect the participants’ ambivalence toward contemplating on the subject or disclosing their personal abusive experience (Malloy et al., 2011; Lev-Wiesel et al, 2018). The use of excessive cuteness or sweetness in drawings as a dissociative mechanism previously was found in various studies that aimed to identify attachment representations in children (e.g., Pianta and Longmaid, 1999; Goldner and Scharf, 2012). Specifically, children characterized by disorganized attachment representation tended to add hearts, flowers, and butterflies to their drawings, and these objects tended to take over much of the page.

The dissociation literature considers dissociation as both a symptom of distress and a defense mechanism that can lead to distress (Nijenhuis et al., 2010; Lahav and Elklit, 2016). The classical approach regards dissociation as a pathological response to trauma in which the victim splits daily reality into the part in which he or she functions appropriately and the abuse experience (Nijenhuis et al., 2010). The two parts of the self, the one that acknowledges the harm and the one that detached itself from it, are thought to be separated on a different level of consciousness (Nijenhuis et al., 2010). However, it is essential to note that using dissociative mechanisms in the narratives and drawings should be regarded as a normative adjustment mechanism since it protects individuals who have undergone painful experiences from emotional flooding (Spiegel et al., 2011; Stein et al., 2013).

Children can be exposed to indirect victimization across multiple socio-ecological contexts, including in the neighborhood, school, and home. Jakob (2018) suggested that even children who do not directly observe domestic or community violence are often aware of violent events or hear repeated accounts of a specific incident and tend to form mental imagery of the violence (Bacchini and Esposito, 2020). In our study, three-quarters of our sample generated narratives of exposure to indirect violence. Data from surveys conducted in the United States showed that more than 58% of the children and adolescents had witnessed or experienced indirect violence, including exposure to war or ethnic conflict, rape, flashing, domestic violence, kidnapping, witnessing a murder, and dating violence (Finkelhor et al., 2007, 2010). Even though the scenes of children who narrated indirect violence narratives tended to involve violence occurring mostly outside the family and their drawings tended to depict less helplessness than those of children who produced narratives of direct exposure to violence, studies have documented harmful effects of indirect exposure to violence, including PTSD symptoms, depression, externalizing problems, and delinquency (Fleckman et al., 2016; Shukla and Wiesner, 2016; Gollub et al., 2019). Thus, the choice to draw indirect violence scenes can attest not only to the high prevalence of the phenomenon in children and adolescents but also to the way children emotionally protect themselves from the consequences of indirect violence and the mental pain involved.

Our study also revealed differences in the children’s drawings and narratives across genders, family structures, and socioeconomic statuses. While boys tended to draw smaller victims and use preschematic drawings, girls produced more vital drawings but negative narratives. These tendencies replicated prior studies that indicated girls’ disposition to depict more expressive metaphorical drawings using a broader range of colors and boys’ tendency to depict more simple and plain drawings (Deaver, 2009; Picard and Boulhais, 2011; Goldner and Levi, 2014). The smaller victim figures in boys’ drawings and the negative narratives of girls can be understood based on boys’ higher rate of physical aggression and girls’ higher rate of emotional aggression, perceived risk of victimization (Carbone-Lopez et al., 2010), and emotional distress (Dao et al., 2006).

In addition, children from families with two parents who were not divorced, and families with higher socioeconomic status tended to use less dissociative mechanisms in their drawings. They drew more abuse scenes (mostly between siblings), and their drawings accorded their narratives. These findings may indicate these children’s higher level of accessibility to the abusive experience and broader internal and external resources to approach the task without bypassing it. Note, however, that other reasons can explain children’s emotional detachment from the drawings, such as their desire to engage with the concept more cognitively and personal predispositions (e.g., personality traits or attachment tendency). Future studies might examine these reasons.

## Conclusion and Limitations

The findings of this study may shed light on the ways children and adolescents perceive child abuse, their concerns, and the ways they try to cope or avoid coping with the distress. Abuse (or a combination of abuse and/or violence, as they perceived it), either within or outside the family, left most of the children feeling lonely and defenseless. Struggling with feelings of humiliation, guilt, and shame, they tried to communicate the abuse/violence, depicting realistic violent scenes, injury, aggressive symbols, and words in an attempt to gain an understanding for the painful experience. Clearly, for some children, art-making accompanied by producing narratives enables a concretization of the abusive experience (Buk, 2009; Loumeau, 2011) as well as its externalization into tangible symbols and narratives, which might support the containment and processing of the event (Avrahami, 2006; Malchiodi, 2012). However, for some children, this task may be perceived as anxiety-provoking and overwhelming, which evokes a more defensive response than would maintaining greater emotional distance (Kaiser, 1996). Since clinicians consider that art-based interventions and drawing techniques facilitate disclosure and foster sharing (Bekhit et al., 2005; Leon et al., 2007), they should be aware of the complexity of asking their clients to produce this type of drawing and the mechanisms children/teens use to cope with evoked distressful feelings.

This study has several limitations that should be taken into consideration. First, the research design was observational and correlative; therefore, it does not allow for causal inference. Second, the study was composed of a small convenience sample of Israeli children and teens who depicted the ways they perceived abuse. Future studies aiming to replicate the findings should apply a representative larger sample. Finally, our findings concentrated on drawings and narrative analyses. Future studies should include additional measures such as self-report questionnaires on children’s adjustment or in-depth interviews to validate the findings and gain a deeper understanding of children’s perceptions of abuse and violence. In addition, personal variables such as the history of trauma and child abuse, emotional regulation, and attachment orientations could also moderate the analysis or shed light on the participants’ perceptions as reflected in their drawings and narratives.

## Data Availability Statement

The raw data supporting the conclusions of this article will be made available by the authors, without undue reservation.

## Ethics Statement

The studies involving human participants were reviewed and approved by the Committee to Evaluate Human Subject Research of the Faculty of Health Sciences and Social Welfare of the University of Haifa. Written informed consent to participate in this study was provided by the participants’ legal guardian/next of kin. Written informed consent was obtained from the minor(s)’ legal guardian/next of kin for the publication of any potentially identifiable images or data included in this article.

## Author Contributions

LG and RL-W analyzed the drawings and data. All authors were involved in writing the article.

## Conflict of Interest

The authors declare that the research was conducted in the absence of any commercial or financial relationships that could be construed as a potential conflict of interest.
